# When Food Safety Goes Viral: The Influence of the Documentary *Poisoned* on Trust in Food Supply Chain Actors and Food Risk Perceptions Among Young Adult College Students in Indiana

**DOI:** 10.1111/1750-3841.71589

**Published:** 2026-10-21

**Authors:** Fanny Gozzi, Shams Adigozalzade, Yaohua Feng

**Affiliations:** ^1^ Department of Food Science Purdue University West Lafayette Indiana USA

## Abstract

As foodborne illnesses pose a significant public health concern in the United States, the rise of streaming platforms broadens audiences’ exposure to food safety documentary films. This investigation examines how the documentary *Poisoned: The Dirty Truth About Your Food* shapes young adults’ perceptions, attitudes, and trust in the food supply chain among undergraduate students at one university in Indiana enrolled in food science (*Study 1, n* = 19) and nonfood science programs (*Study 2*, *n* = 59). An explanatory mixed‐methods design combined surveys at three time points with a focus group discussion. Surveys assessed perceptions of food safety importance and confidence in food supply actors using Wilcoxon signed‐rank tests, while focus group transcripts were analyzed thematically. Participants reported strong emotional responses, including fear, frustration, and distrust toward the food industry and regulatory bodies, while describing the documentary as informative and effective in raising awareness about foodborne illness risks and the importance of food safety practices. Differences emerged by educational background, with food science majors demonstrating more critical engagement, whereas some nonfood science participants reported intentions to adopt risky food‐handling practices, such as washing raw poultry. Confidence in food supply chain actors declined significantly after viewing. At 9‐day follow‐up, food science students regained trust, while nonfood science students showed recovery only toward consumer‐facing actors and food they prepared themselves. These findings suggest that emotionally compelling documentaries can increase food safety awareness and engagement while reducing trust and unintentionally reinforcing unsafe practices. Rather than serving as educational tools, such documentaries offer valuable lessons for improving transparent, accurate, and balanced food safety communication.

**Practical Applications:**

This research offers food industry, government agencies, educators, and media creators with insight into how the documentary *Poisoned* can influence young adults' in college perceptions and responses to food safety issues. Since documentaries are widely available and can reach broad audiences, findings highlight the importance of proactive, transparent, and collaborative communication, helping audience understand how the food system works, how food safety hazards are addressed, and what can be done in the kitchen to reduce risks. Cross‐sector collaboration that combines engaging storytelling with reliable educational content may improve public understanding, strengthen confidence in the food system, and encourage safer food‐handling practices.

## 1 Introduction

Food safety remains a major public health concern in the United States, with an estimated 48 million Americans experiencing foodborne illnesses each year (CDC 2025). Beyond the direct health burden, those illnesses also erode public confidence in the systems designed to ensure a safe food supply. Frequent foodborne outbreaks and product recalls exacerbate this erosion, especially when consumers perceive insufficient transparency or accountability (IFIC 2025). Effective communication strategies are therefore essential for improving public understanding of food safety risks. Short video and films have become an important means of shaping public awareness and understanding, serving as an effective tool for providing food safety information (Majestya and Irwanto 2025; USDA FNA 2025). By combining factual information with emotionally engaging narratives, media storytelling may influence public perceptions and motivate behavioral change (Shaffer et al. 2018). Documentaries represent one such format, presenting complex issues through real‐world events and personal experiences. Research has shown that audiovisual learning formats capture viewers’ attention and make complex material easier to understand and retain (Shabiralyani et al. 2015).

Based on Jeff Benedict's book *Poisoned: The True Story of the Deadly E. Coli Outbreak That Changed the Way Americans Eat*, Stephanie Soechtig's documentary *Poisoned: The Dirty Truth About Your Food*, released in 2023 on Netflix, sparked public debate about food safety failures across the US food supply chain (Henley 2025). The film traces the history and consequences of major US foodborne illness outbreaks through interviews with food safety experts, food industry representatives, food safety advocates, and testimonies from victims and their families (Soechtig 2023). The documentary encourages questioning whether current industry practices and regulatory systems adequately protect US consumers. Soechtig is a documentary filmmaker known for investigative films addressing public health, US food systems, and corporate accountability, including Fed Up (2014) and Under the Gun (2016). Like many documentary films intended for broad audiences, *Poisoned* also relies on narrative, editorial, and emotional framing that may shape viewers’ interpretations and perceptions of the food‐safety issues presented. These characteristics highlight the importance of considering both the potential educational value and the limitations of documentary films as a source of public information.

As a widely accessible documentary distributed through a major streaming platform, *Poisoned* offers a relevant example of how food‐safety issues may be communicated to the public outside traditional educational settings. Established food safety education strategies, including in‐person workshops (Chen et al. 2023), community outreach programs (Yeung et al. 2019; Berejena and Malongane 2025), or online learning modules (Berglund et al. 2024), have been well studied as methods for improving consumers’ understanding of food safety practices and risk prevention. However, documentaries may offer a distinct approach by integrating informational content with emotionally engaging audiovisual storytelling, which has the potential to shape viewers' food safety perceptions (Uhrig et al. 2016). Yet comparatively little is known about the potential impact of documentary films such as *Poisoned* on viewers’ perceptions and confidence related to food safety and food supply chain actors.

Although studies on documentary and narrative media are limited, existing research suggests that documentaries can influence viewers’ attitudes and emotional responses. McKay et al. (2022) reported that a documentary on wheelchair basketball positively affected college students’ attitudes and actions toward inclusion. Similarly, Boissat et al. (2021) reported that *Blackfish*, a documentary addressing marine mammal captivity, was associated with shifts in public attitudes, broader behavioral, and societal changes concerning marine mammal entertainment in parks such as SeaWorld. These findings highlight that documentary‐based communication may serve as an effective educational tool by combining emotional impact with evidence‐based content. This raises an important question in the context of food safety: can documentaries distributed through widely accessible streaming platforms similarly influence how viewers perceive food safety and the major actors of the food supply chain?

Young adults represent an important population for examining this question. Individuals aged 18–34 years old are among the most frequent users of streaming media platforms, with approximately 75% of individuals in this age group having access to a Netflix subscription, increasing the likelihood of exposure to documentary content (Statista 2021). Documentary films also have been recognized as useful instructional resources in university settings, supporting student engagement with complex scientific and societal issues (Kankal et al. 2023). This is particularly relevant for young adults, as this life stage represents an important window for shaping how individuals understand food‐related risks and make decisions about the foods they obtain, prepare, and consume (Tam et al. 2017). University students are an especially important population to consider, as previous studies have identified gaps in food safety knowledge and practices among the college‐aged population (Lin 2013; Chuang et al. 2021; Chen et al. 2024). In addition, students may differ in their prior exposure to food safety information depending on their academic background, making it important to examine both food science and nonfood science majors. Prior knowledge may influence how audiences interpret and respond to documentary content, as existing literature suggests that it can shape the relationship between attitude, intentions, and subsequent behavior (Gugenishvili and Laine‐Kronberg 2025).

Accordingly, the present work focused on examining how the documentary *Poisoned* influenced two groups of undergraduate students at a public university in Indiana: students enrolled in food science and nonfood science programs. The study focused on their awareness of food safety and their confidence in eight key actors across the food supply chain: small‐ and large‐scale farmers, small‐ and large‐scale food processors, US government regulators, grocery retailers, restaurants, and consumers. Specifically, this analysis explored whether exposure to the documentary *Poisoned* was associated with changes in interest in learning about food safety, emotional responses to food‐related risks, and confidence in key actors across the food supply chain. It also considered potential documentary bias, emotional framing, or exaggeration that could shape the viewer's interpretation of the presented information. In doing so, this work provides preliminary insight into both the potential and limitations of a documentary film such as *Poisoned* for engaging audiences with food safety issues, using college students at a public university as an example.

## 2 Materials and Methods

### 2.1 Study Design

This study was conducted using an adapted mixed‐methods explanatory sequential design (Ivankova et al. 2006), combining quantitative and qualitative data. This methodology was chosen to generate complementary data on the same topic to provide a more comprehensive interpretation of the findings (Morse 1991). Quantitative and qualitative data were collected sequentially, analyzed separately, and merged together for results (Figure 1).

**FIGURE 1:**
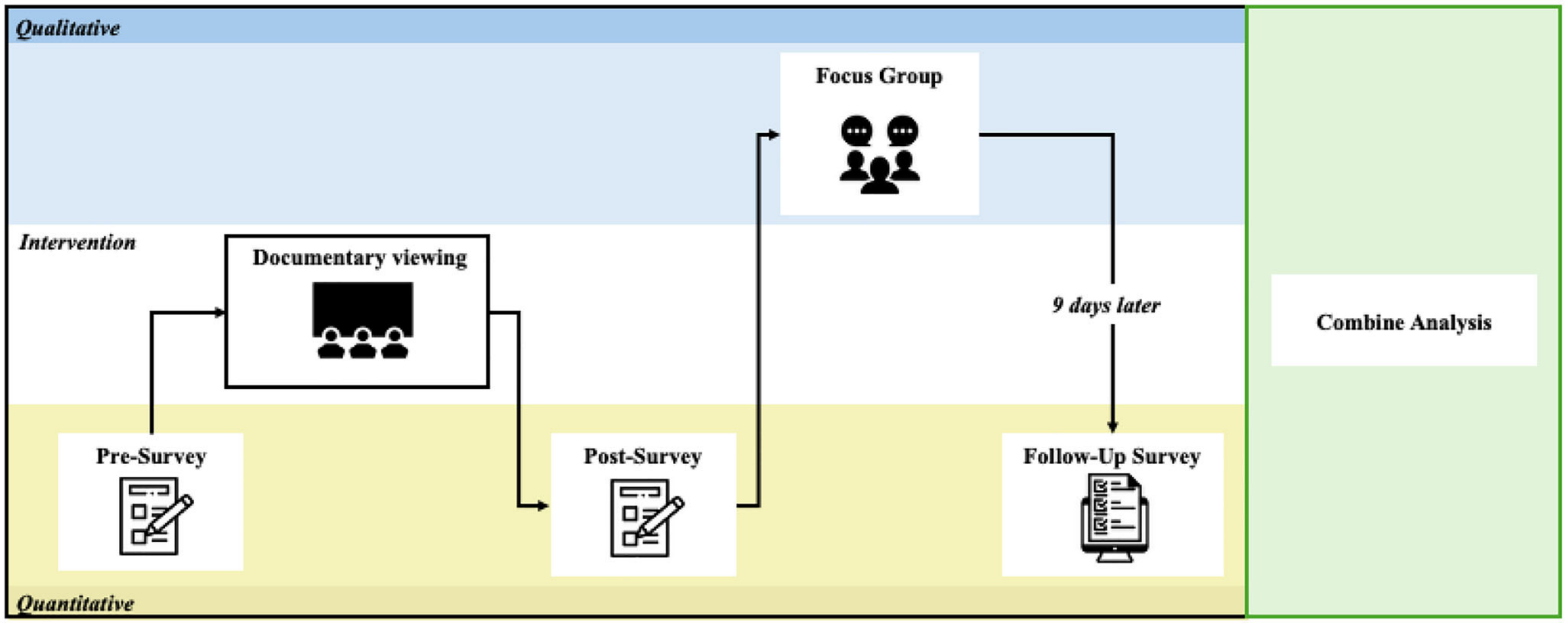
Overview of the adapted explanatory sequential mixed‐methods study (Ivankova et al. 2006).

*Quantitative data collection* followed a structured timeline: participants first completed a pre‐survey, then watched the documentary *Poisoned: The Dirty Truth About Your Food*, followed by an immediate post‐survey. A follow‐up survey was administered online 9 days later via Qualtrics (Provo, UT) (Cottone and Byrd‐Bredbenner 2007). Surveys aimed to measure changes in participants’ trust levels toward eight specific actors in the food supply chain, as well as the importance they placed on the safety of the foods they represented.

*Qualitative data collection* took place directly after the post‐survey during the same session. Participants engaged in a focus group discussion exploring their interpretations of the documentary, the reasoning behind any changes in their survey responses, and the potential increase in knowledge resulting from the intervention. This adapted sequential design allowed the researchers first to quantify changes following the documentary intervention and then to explore the underlying reasons, interpretations, and personal reflections behind those changes through qualitative insights gathered during the focus group. This study design enabled a more comprehensive understanding of the potential impact of the documentary on young adult viewers.

All sessions were conducted in person and were scheduled based on university room availability and considering participants’ schedules to maximize convenience. Researchers also made sure participants with identical first names were placed in different groups to minimize confusion during audio transcription. Groups were intentionally organized to maintain diversity in participants’ academic majors and gender. All participants provided written informed consent prior to their participation. Pre‐ and post‐surveys were completed using paper‐and‐pencil and later digitized by a researcher and checked by the main investigator. This paper‐and‐pencil approach allows participants to reflect on their responses and discuss changes during the focus group. The survey (S2) and focus group (S3) scripts were reviewed and revised based on feedback from internal food safety experts, enhancing validity and clarity. The research protocol was reviewed by the Institutional Review Board of Purdue University (IRB #2024‐1158) prior to data collection.

#### 2.1.1 Study 1: Food Science Students

In August 2024, *Study 1* was conducted using convenience sampling to recruit undergraduate students enrolled in a food science course at a large public university in Indiana. A total of 23 student participants were divided into four focus groups. Four follow‐up surveys were not returned, so their data were excluded from the analysis, yielding a final sample of 19. One participant did not speak during the focus group session but was still included in the analysis, because silent participants most often align with or endorse group sentiment through nonverbal cues or implicit agreement (Onwuegbuzie et al. 2009). Additionally, this initial study provided baseline data to examine potential differences between students majoring in food science and those from other academic disciplines.

#### 2.1.2 Study 2: Nonfood Science Students

*Study 2* took place between October and November 2024. Samples included undergraduate students enrolled in a nonfood science program at a large, 4‐year public university in Indiana. Researchers recruited participants using a convenience sampling approach, supplemented by snowball sampling. Recruitment efforts included posting physical flyers at designated campus locations, outreach via social media, and participant referrals. Interested individuals completed an online screening questionnaire (S1) via Qualtrics (Provo, UT). College students were specifically targeted based on literature demonstrating that 75% of individuals aged 18 to 34 in the United States have a Netflix subscription (Statista 2021) and recognizing that this age group represents a critical developmental period for establishing long‐term food consumption and purchasing behaviors (Tam et al. 2017). The sampling strategies were designed to support an exploratory mixed‐methods investigation of university‐enrolled young adults (18–34 years old) responses to the documentary rather than to generate a population estimate representative of all the US young adults. In Study 2, of the 192 individuals who initiated the recruitment survey, only 144 provided complete responses (42 did not provide their email addresses, and six omitted required questions). Of these respondents, 90 confirmed participation and scheduled a session. Ultimately, the final sample consisted of 59 participants (representing a 34.5% attrition rate) organized into 10 focus groups, averaging approximately six participants per group. Smaller focus groups offer more comfort to the participants (Krueger and Casey 2015). Although the original design intended to include participants who had previously viewed the documentary along with first‐time viewers, only two individuals from the former group consented to participate. Consequently, all focus groups comprised exclusively first‐time viewers, ensuring consistency across the sample.

### 2.2 Quantitative Analysis

To assess participants’ perceptions of food safety and trust in various food supply chain actors, data collection occurred at three time points surrounding the intervention. The session began with participants completing a paper‐based pre‐survey, which served to establish baseline confidence levels in eight actors within the food supply chain: small‐scale farmers, large‐scale farmers, small‐scale processors, large‐scale processors, grocery stores, restaurants, US government representatives, and food that I cook myself. Confidence in each actor was measured by asking participants how confident they were in that actor's ability to produce food that would not cause illness, using a 7‐point Likert scale (1 = *strongly disagree*, 7 = *strongly agree*). In addition, the perceived importance of food safety was measured using a 5‐point Likert scale (1 = *not at all important*, 5 = *very important*). All the moderators were trained to provide clarification or definitions of the actors presented upon participants’ request; however, no participants in either study requested additional information. Immediately following the pre‐survey, participants viewed the documentary. After viewing, participants completed the paper post‐survey, identical content to the pre‐survey, which measured any immediate changes in their levels of trust. Nine days later, participants were contacted by email and asked to complete an online follow‐up survey in Qualtrics (Provo, UT). The final survey, identical to the previous instruments, was designed to evaluate the persistence of any changes over a short period of time. Internal consistency of the confidence scale was evaluated using Cronbach's alpha, yielding acceptable reliability for nonfood science participants pre‐survey (*α* = 0.790), and high reliability for post‐survey (*α* = 0.869) and follow‐up survey (*α* = 0.863). For food science participants, the pre‐survey (*α* = 0.894), post‐survey (*α* = 0.909), and follow‐up survey (*α* = 0.859) were in accordance with established guidelines (Taber 2018). In addition, descriptive statistics and Wilcoxon signed‐rank tests using SPSS (IBM Corp., Armonk, NY) were conducted to identify significant changes in participants’ confidence levels across the three surveys (Wilcoxon 1945).

### 2.3 Qualitative Analysis

To provide context for qualitative findings and achieve a deeper understanding of participants’ perceptions, qualitative data were collected through a semi‐structured, open‐ended focus group conducted immediately after participants completed the post‐survey. This approach enables richer interpretation and facilitates understanding of observed trust shifts. Each focus group was moderated by a trained member of the Purdue University Food Safety Human Factor laboratory research team using the previously revised and reviewed script. All sessions were conducted in English, with an average duration of 48 min. To ensure accuracy, focus group discussions were audio‐recorded and transcribed verbatim using Otter.ai transcription software (Mountain View, CA). Transcripts were subsequently reviewed and manually verified by the research team to correct errors and preserve clarity and meaning. Qualitative data were analyzed using Braun and Clarke's thematic analysis method (Braun and Clarke 2006), a robust and adaptable framework well‐suited to reveal underlying themes and patterns in qualitative data. NVivo 14 software (Denver, CO) was used to facilitate data organization, coding, and analysis. Throughout the analytics process, the primary author and research assistant independently coded the qualitative data and developed the initial codebook. Areas of agreement and divergence were then discussed collaboratively, with codes reviewed and refined for clarity and consistent application across the focus group dataset. When discussion could not resolve interpretation differences, a senior research team member with expertise in qualitative data analysis was consulted to provide an additional perspective and support resolution. The codebook was refined using this process, and the principal investigator reviewed the final coding framework for consistency and validity of the interpretations.

## 3 Results and Discussion

### 3.1 Demographics

A total of 78 undergraduate students participated across the two studies (*Study 1*: *N* = 19, *Study 2*: *N* = 59) following the protocol described in Figure 1. The majority of the participants were aged 18‐ to 24‐year‐olds and were females—74% in *Study 1* and 78% in *Study 2* (Table 1). In *Study 1*, all students were food science majors enrolled in a required food sanitation course and were juniors (11%, *n* = 2) or seniors (89%, *n* = 17). In contrast, *Study 2* included a more diverse group of majors excluding food science students: 24% from the College of Engineering, 20% from Health and Human Sciences, 20% from the College of Science, 12% from the School of Business, and smaller proportions from the Polytechnic Institute, Liberal Arts, Agriculture, and Pharmacy. Participants represented all college class levels, including freshmen (34%, *n* = 20), sophomores (29%, *n* = 17), juniors (25%, *n* = 15), and seniors (12%, *n* = 7). *Study 2* also captured a broader range of participants’ demographics than the work with food science students, including ethnicity and cooking frequency, with 46% identifying as White, non‐Hispanic, and 42% reporting cooking meals at least three times a week.

**TABLE 1:** Demographic characteristics of participants for Studies 1 (food science) and 2 (nonfood science).

Characteristics	Study 1 (*N* = 19)		Study 2 (*N* = 59)	
Gender				
Male	5	26%	13	22%
Female	14	74%	46	78%
Age				
18–24 years	19	100%	58	98%
25–34 years	0	0%	1	2%
College major				
Engineering	0	0%	14	24%
Health and Human Science	0	0%	12	20%
Science	0	0%	12	20%
School of Business	0	0%	7	12%
Polytechnic Institute	0	0%	5	8%
Liberal Art	0	0%	4	7%
Agriculture	19	100%	3	5%
Pharmacy	0	0%	2	4%
Grade				
Freshman	0	0%	20	34%
Sophomore	0	0%	17	29%
Junior	17	89%	15	25%
Senior	2	11%	7	12%
Ethnicity				
White (non‐Hispanic)	—		27	46%
Asian	—		22	37%
Hispanic	—		5	8%
Other	—		3	5%
Black or African American	—		1	2%
Prefer not to answer	—		1	2%
How often have you cooked in the last 6 months?				
I cook once a week.	—		14	24%
I cook about 3–4 times a week.	—		13	22%
I've cooked almost every day.	—		12	20%
I've only cooked a couple of times in the last 6 months.	—		10	17%
I've cooked a few times a month.	—		9	15%
I haven't cooked at all in the last 6 months.	—		1	2%

### 3.2 Documentary Storytelling Drawbacks: Emotions and Confidence Impact

#### 3.2.1 Emotional Responses to the Documentary

The documentary *Poisoned* prompted strong emotional engagement among nonfood science major young adult participants, with post‐viewing discussion revealing that firsthand testimonies of foodborne illness made the issue feel deeply personal and emotionally impactful. Participants’ responses were reflected in a variety of emotions during the focus groups, with several of them sharing that the personal stories moved them to tears. One participant explained, “*[…] I cried several times during this, not going to lie, like it was really emotional, and it was hard to watch, like, when you see all that happening to those families*” (FG1 P5 S2). This suggests that the documentary's use of personal narratives effectively humanized the consequences of foodborne illness and increased emotional engagement with the topic. The observed changes in participants’ emotional responses are consistent with research showing that narrative structures, personal testimonies, and visual storytelling can evoke strong emotional responses and increase audience identification with the subject matter (Bondebjerg 2014; Or et al. 2023).

Among the emotions expressed during the study, fear was most commonly reported by participants. For nonfood science participants, the terms “fear” or “scared” and their declensions appeared across the 10 groups, reflecting participants’ concerns regarding food safety risks and the emotion the documentary evoked in viewers. Certain participants explain their fear as arising from uncertainty about the safety of foods purchased from grocery stores, as illustrated by one participant who stated, “*I thought it made me kind of feel scared and, like, made me kind of want to check the recall list and make sure I'm not, like, buying food that is not good. […]*” (FG8 P47 S2*)*. Another described becoming more apprehensive of the food served at the University: “*Now I'm scared of eating lettuce in the dining hall and, yeah, so like, it made me, like, more aware of the risk of food, which is a big concern*” (FG9 P50 S2). It also extended beyond specific foods or settings, with participant 43 expressing that “*[…] a lot of it is kind of like, makes you fearful of what you like eat*” (FG8 P43 S2). The term “fear mongering” was also used by participants to characterize the perceived intent and tone of the documentary: “*I feel like the documentary at least made me feel like it was fear‐mongering a little bit for me, because I feel like a lot of people who are uneducated can watch that it's very easily accessible, and it scares you a lot*” (FG4 P23 S2). This aligns with research showing that documentary storytelling and emotionally compelling narrative can evoke strong emotional responses such as fear, making health or societal risks feel immediate and personally relevant (Bieniek‐Tobasco et al. 2019). For some participants, this translated into self‐reported increased vigilance “*I'm gonna be like, be careful about what you're eating when you when you're going out. Be careful that, you know, don't ask for medium rare or whatever. Just ask the perfect temperature and stuff*” (FG10 P57 S2), and a greater sense of personal importance of food‐handling practices at home “*I can try to, you know, wash my hands and not cross‐contaminate and wash everything*” (FG6 P32 S2). Health related documentary films incorporating real‐life testimonies may be perceived as more credible, fostering empathy among viewers (Daugelat et al. 2024). Existing literature has also shown that emotional engagement and personal reflection can lead viewers to express stronger intentions to adopt protective health behaviors (Mubangizi et al. 2022). Participants in the present study may have perceived the risks as more personally relevant through their emotional connection to the individual portrayed in the documentary, fostering greater self‐reported awareness and responsibility regarding food safety practices.

This reaction to the film also led to increased worry and anxiety of participants about their own refrigerators and food handling practices “*[…] I just bought some chicken from Target. And I'm like, wow, like, this has Salmonella, I don't know, like the points of contamination on, like, cooking it. I'm like, I have to be really careful now*” (FG2 P12 S2). Beyond the concerns of purchasing and food handling practices, responses also showed worries regarding having children in the future after hearing parents' testimonies of losing a child due to foodborne illness, “*I'm not like worried for myself. It made me scared to have kids in the future*” (FG5 P25 S2). These responses suggest that the documentary made food safety risks feel more personal and immediate. Participants appeared to distinguish between risks within their personal control and those managed by food supply chain actors. While emotional responses increased vigilance and a sense of importance for food‐handling practices at home, frustration and distrust were directed toward the food industry and government regulators perceived as responsible for preventing such risks. Emotional storytelling appeared to deepen engagement; at the same time, emotionally charged narratives and audiovisual information may also shape perceptions in ways that extend beyond the intended educational message, including heightened distrust of actors across the food supply chain (Konijn et al. 2009).

Frustration and distrust were expressed toward some key actors in the food supply chain, mainly criticizing both the food industry (perceived as profit‐driven and negligent) and the governmental regulatory bodies (perceived as ineffective or passive). “*The industries aren't going to do it out of the goodness of their hearts. They're going to do whatever makes the most money. So obviously the government needs to, like, set rules so that they're forced to do it. And so, they're like, oh, you can do it, but you don't have to*” (FG1 P7 S2). Distrust in the food industry appeared to be connected to the perception of companies prioritizing profit over public health. The frustration and distrust expressed by participants also are consistent with broader survey findings indicating low public confidence in industry and regulators when food safety is perceived to be compromised (IFIC 2025).

Similar emotional reactions toward the documentary were reported by participants enrolled in a food science degree (*Study 1*). Participants again described feelings of fear following the viewing. For example, “*I am so scared I have another bag of, like, salad mix in my fridge right now*.” (FG3 P16 S1), while another reflected, “*I was scared. [talking about viewing the documentary] I was just thinking of like, everything that I've eaten and like, if, like it was possible it was contaminated somehow*.” (FG2 P11 S1) with participant 7 following up by “*A little bit angry*” (FG2 P7 S1). In this study, participants’ expressions of distrust reflect concern about the level of trust placed in actors of the food supply chain, as illustrated by one participant: “*I agree also, consumers, like are putting so much trust in these companies, but like, they really probably shouldn't*” (FG2 P9 S1). However, they demonstrated greater caution in their documentary interpretation, suggesting that using editing effects to deliver a message does not fully capture the complexity of the issue for the audience. “*My feelings are pretty conflicted, because on one hand, like, obviously, like the personal anecdotes and the stories they shared of people […] I felt sad for them, sympathy for them. But I also like approach things like this with caution and kind of, like, skepticism, especially when it's presented in a way that's very like, one‐sided, because clearly, like, they made edits and stuff to like, have people repeating themselves, or like, they clearly displayed all of the funding that they got from research. […]*” (FG2 P8 S1). Food science participants were more likely to question whether the film presented only one side of the story and evaluated the documentary more critically, while considering the potential lack of context regarding the food industry perspective. The contrast between food science and nonfood science students is particularly notable. Although both groups responded emotionally, food science students appeared more likely to maintain critical distance and recognize the possibility of selective framing or oversimplification. This finding suggests that prior knowledge may moderate emotional engagement, enabling viewers to interpret documentary content more analytically rather than relying primarily on emotional cues (Bondebjerg 2014). Taken together, these results indicate that documentary storytelling can be highly effective in drawing attention to food safety and food supply chain issues, while provoking strong emotional responses, although its effects may differ depending on the viewer's background and familiarity with the topic.

#### 3.2.2 Confidence Shifts Toward Major Food Supply Chain Actors

To further evaluate the changes in participants' confidence, responses from the surveys were analyzed and compared to examine the potential impact of the documentary on food supply chain actors. Significant declines in confidence across all food supply chain actors were observed between pre‐ and post‐survey results (Table 2), indicating reduced trust in the food supply chains immediately following documentary viewing.

**TABLE 2:** Results from Wilcoxon signed‐rank test results for pre‐ and post‐intervention measures for Study 1 and Study 2.

	Pre survey		Post survey		Wilcoxon *Z*	*p*	*r*
	*M* ± SD	Md	*M* ± SD	Md			
Study 1 (*n* = 19)							
US government	5.316 ± 1.293	6	3.211 ± 1.032	3	−3.771	***	0.865
Small farmers	5.000 ± 0.816	5	3.684 ± 1.336	4	−3.698	***	0.848
Large farmers	4.842 ± 1.015	5	2.842 ± 1.259	3	−3.867	***	0.887
Small processors	5.105 ± 0.937	5	3.684 ± 1.416	4	−3.582	***	0.822
Large processors	5.158 ± 1.068	5	3.053 ± 1.393	3	−3.775	***	0.866
Restaurants	4.684 ± 1.293	5	3.684 ± 1.293	4	−2.695	**	0.618
Grocery stores	5.053 ± 1.079	5	3.421 ± 1.346	3	−3.680	***	0.844
Myself	5.211 ± 1.032	5	4.158 ± 1.385	4	−2.981	**	0.684
Study 2 (*n* = 59)							
U.S. government	4.186 ± 1.196	4	2.305 ± 1.038	2	−6.474	***	0.843
Small farmers	5.220 ± 1.001	6	3.610 ± 1.246	3	−5.755	***	0.749
Large farmers	4.373 ± 1.202	4	2.203 ± 1.047	2	−6.628	***	0.863
Small processors	4.814 ± 1.167	5	3.407 ± 1.146	3	−5.617	***	0.731
Large processors	4.102 ± 1.255	4	2.102 ± 1.094	2	−6.436	***	0.838
Restaurants	4.407 ± 1.328	5	2.831 ± 1.132	3	−5.926	***	0.771
Grocery stores	5.085 ± 0.915	5	2.966 ± 1.082	3	−6.579	***	0.857
Myself	5.932 ± 0.785	6	4.712 ± 1.246	5	−5.457	***	0.710

*Note*: Participants rated their agreement with each statement on a 7‐point Likert scale (1 = *strongly disagree*, 7 = *strongly agree*). Medians are reported for Wilcoxon signed‐rank test, a nonparametric method that evaluates differences in ranked observations rather than mean differences. Study 1: Participants enrolled in a food science bachelor's degree program; Study 2: Participants enrolled in bachelor's degree programs other than food science.

Abbreviations: *M* ± SD, mean ± standard deviation; Md, median; *p*, *p*‐value; *r*, effect size.

***p < 0.001; **p < 0.01; *p < 0.05.

Among food science participants, confidence declined across all eight evaluated actors, with the largest reduction observed for large‐scale farmers. Mean confidence ratings decreased from *M* = 4.84 (SD = 1.02; Md = 5) prior to viewing to *M* = 2.84 (SD = 1.26; Md = 3) following documentary viewing (*r* = 0.89; *p* < 0.001), representing a substantial decline in participants’ confidence. Similar reductions were observed for US government regulators and large‐scale food processors (Table 2), suggesting that participants perceived institutional and large‐scale actors as less capable of ensuring food safety after viewing the documentary. Nonfood science participants demonstrated an even stronger pattern of diminished trust, with confidence in large‐scale farmers again showing the most important declines, decreasing from *M* = 4.37 (SD = 1.20; Md = 4) to *M* = 2.20 (SD = 1.05; Md = 2) (*r* = 0.86; *p* < 0.001), with similarly pronounced reductions observed for US government regulators and grocery stores (Table 2). The large Wilcoxon effect sizes, together with notable reductions in confidence scores, indicate a consistent and meaningful decrease in confidence across the actors of the food supply chain presented (Cohen 1988). Qualitative responses provided further context for these findings. Several students questioned the effectiveness of US food regulatory oversight and contrasted it with European systems presented in the documentary with stricter food safety standards. As one participant stated, “*My score decreased because they're really not doing as much as they should be. Like, I mean, you look at Europe, they cut their Salmonella rates in half. Why can't we do that?*” (FG10 P55 S2). This pattern suggests that participants interpreted *Poisoned* not simply as highlighting isolated foodborne illness incidents, but as exploring broader perceived weaknesses. Consistent with this interpretation, the observed reductions in confidence across both studies suggest that viewing the documentary immediately diminished trust in food supply chain actors. Participant concerns align with broader literature indicating the fragility of public trust in large food manufacturers and retailers (Mosier 2023; Dilley et al. 2026). Students emphasized that “basic food safety should be a given,” not something consumers should need to manage at every stage of food preparation. This perspective is consistent with research showing that consumers increasingly value transparency in the food industry and tend to place greater trust in companies and brands that provide clear information about their production processes, even when that information triggers uncertainty (Jaeger et al. 2015; Bruhn and Feng 2021; Feng and Bruhn 2025). The declines in confidence observed across both studies, particularly for large‐scale farmers and processors, and the US government regulators, suggest that participants interpreted the documentary as emphasizing broader concerns related to US food safety oversight. Skepticism also extended to the US regulatory environment, which participants perceived as less rigorous than their European counterparts (Wagenberg et al. 2012; Bánáti and Baicu 2024).

Despite this overall decrease, home‐prepared food remained the most trusted category among participants in both studies. Although confidence in participants’ own food preparation also decreased following documentary viewing, the reductions were comparatively modest. In *Study 1*, confidence declined from *M* = 5.21 (SD = 1.03; Md = 5) to *M* = 4.16 (SD = 1.39; Md = 4) (*r* = 0.68; *p* < 0.01), while in *Study 2*, scores decreased from *M* = 5.93 (SD = 0.79; Md = 6) to *M* = 4.71 (SD = 1.25; Md = 5) (*r* = 0.71; *p* < 0.001). Although participants reported increased awareness of food safety risks following exposure to the documentary, they continued to express relatively greater confidence in food they prepared themselves. Home‐prepared food remained the most trusted source in both studies, despite a modest decline in confidence following the viewing. This pattern may reflect a perceived sense of control over personal food preparation, even as the documentary brought awareness on household food safety risks, such as cross‐contamination, encouraging them to reflect on their own food‐handling practices and recognize potential failures. Prior research from IFIC similarly indicates that 66% of survey respondents believe that they have a greater sense of control over the food prepared in their homes (IFIC 2020). IFIC Food & Health Survey of 2025 also indicates that 59% of respondents lack confidence in food supply chains because they believe that profit is prioritized over safety in the industry (IFIC 2025). The documentary may therefore have reinforced participants' skepticism toward large institutional actors perceived as primarily responsible for preventing contamination of food.

After assessing immediate changes in confidence from pre‐survey to post‐survey, follow‐up responses collected 9 days later were analyzed to determine whether these changes persisted over time (Table 3). For food science students, a significant increase in confidence across seven of the eight actors presented was observed between the immediate post‐viewing survey and follow‐up (*p *< 0.05), indicating partial confidence recovery over time. Confidence in restaurants’ ability to provide safe food increased, though the change was not statistically significant. Participants enrolled in food science majors voiced particular opinions about food safety practices in restaurants, noting that poor handling could directly affect their business “*[…] I feel like restaurants, if they don't serve good food, and they don't make the food safe for consumers, they're not going to have people to come back to eat their product […]*” (FG5 P5 S1). In contrast, food directly cooked in participants’ home kitchens observed the most important rebound in confidence, which rose from a mean of *M* = 4.16 (SD = 1.39; Md = 4) immediately after viewing to *M* = 5.00 (SD = 2.76, Md = 5) at follow‐up (*r* = 0.63; *p* < 0.01). Nonfood science students showed a very different pattern, with a more limited recovery in confidence. A significant increase in confidence between the post and follow‐up surveys was found in three of eight actors, with restaurants increasing from *M* = 2.83 (SD = 1.13, Md = 3) to *M* = 3.29 (SD = 1.19; Md = 3) (*r* = 0.48; *p* < 0.001), grocery stores from *M* = 2.97 (SD = 1.08, Md = 3) to *M* = 3.39 (SD = 1.15; Md = 3) (r = 0.38; *p* < 0.01), and food that participants cook themselves from *M* = 4.71 (SD = 1.25; Md = 5) to *M* = 5.03 (SD = 1.14; Md = 5) (*r* = 0.28; *p* < 0.05).

**TABLE 3:** Results from Wilcoxon signed‐rank test results for post‐ and follow‐up intervention measures for Study 1 and Study 2.

	Post Survey		Follow‐up Survey		Wilcoxon *Z*	*p*	*r*
	*M* ± SD	Md	*M* ± SD	Md			
Study 1 (*n* = 19)							
US government	3.211 ± 1.032	3	3.789 ± 1.134	4	2.057	*	0.472
Small farmers	3.684 ± 1.326	4	4.368 ± 1.116	4	2.032	*	0.466
Large farmers	2.842 ± 1.259	3	3.474 ± 0.905	3	2.08	*	0.477
Small processors	3.684 ± 1.416	4	4.316 ± 1.204	4	2.025	*	0.465
Large processors	3.053 ± 1.393	3	3.789 ± 1.084	4	2.496	*	0.572
Restaurants	3.684 ± 1.293	4	4.000 ± 1.054	4	0.823		0.189
Grocery stores	3.421 ± 1.346	3	4.263 ± 0.991	4	2.556	*	0.586
Myself	4.158 ± 1.385	4	5.000 ± 2.757	5	2.757	**	0.632
Study 2 (*n* = 59)							
US government	2.305 ± 1.038	2	2.492 ± 1.150	2	1.684		0.219
Small farmers	3.610 ± 1.246	3	3.847 ± 1.096	4	1.704		0.222
Large farmers	2.203 ± 1.047	2	2.271 ± 0.944	2	0.692		0.090
Small processors	3.407 ± 1.146	3	3.441 ± 1.055	3	0.239		0.031
Large processors	2.102 ± 1.094	2	2.136 ± 0.955	2	0.339		0.044
Restaurants	2.831 ± 1.132	3	3.288 ± 1.190	3	3.720	***	0.484
Grocery stores	2.966 ± 1.082	3	3.390 ± 1.145	3	2.905	**	0.378
Myself	4.712 ± 1.246	5	5.034 ± 1.144	5	2.150	*	0.280

*Note*: Participants rated their agreement with each statement on a 7‐point Likert scale (1 = *strongly disagree*, 7 = *strongly agree*). Medians are reported for Wilcoxon signed‐rank test, a nonparametric method that evaluates differences in ranked observations rather than mean differences. Study 1: Participants enrolled in a Food Science Bachelor's degree program; Study 2: Participants enrolled in Bachelor's degree programs other than food science.

Abbreviations: *M* ± SD, mean ± standard deviation; Md, median; *p*, p‐value; *r*, effect size.

***p < 0.001; **p < 0.01; *p < 0.05.

The observed findings suggest that participants showed a tendency to regain confidence in the food supply chain actors over time, particularly for actors with whom they commonly interact or perceive greater personal control. However, despite the partial recovery, confidence levels at the 9‐day follow‐up generally remained below pre‐viewing survey baseline levels, indicating that the documentary's influence on participants’ confidence in the safety practices by food supply chain actors was not entirely transient. This pattern is consistent with broader national trends reporting a decline in US consumer confidence in the safety of the food supply, particularly toward food manufacturers, US regulators, and retailers (IFIC 2025). Differences between the two study populations may reflect variations in prior food safety knowledge and educational background, particularly given the differences in participants’ level of education and academic major. Food science students regained their confidence with significant increases across seven of eight food supply chain actors. Their academic background in microbiology, food science, and food regulations appeared to help them understand the explanations behind the scary headlines, which may have lessened their emotional response to the documentary and made it feel more like an educational resource about current issues. The existing literature shows that students’ food safety knowledge and educational exposure affect their knowledge and can shape their attitudes toward food safety (Marklinder et al. 2022).

In contrast, nonfood science participants in *Study 2* exhibited a more limited recovery overall, with significant confidence regain observed for only three of the eight actors. However, restaurants showed the strongest rebound among nonfood science students, suggesting that participants without specialized food safety education may have been more willing to regain confidence in food service settings over time. A different pattern emerged in *Study 1*, in which restaurants appeared to be the only actors that did not show a statistically significant increase between the post‐ and follow‐up surveys. As reflected in the focus group findings, these participants appeared to maintain greater skepticism toward restaurant food safety practices. Kramer et al. (2023) emphasize that a strong food safety culture in restaurants depends on managerial commitment and adequate resources. Given food science participants’ educational background, they are likely to be more aware of systematic problems in restaurant hygiene, handling, and compliance.

Additionally, in both studies, food prepared by participants themselves maintained the highest confidence ratings and demonstrated a strong recovery over time. This reflects consumers’ greater trust in food sources that they perceive as having a higher degree of personal control, such as food prepared in their own home. They may feel more confident when they can directly influence food safety through choosing ingredients in grocery stores, cooking properly, and keeping things clean (IFIC 2025). This aligns with broader food risk perception research, which shows that consumers tend to evaluate risks differently depending on how controllable or familiar the food source appears. Risks perceived as voluntary, familiar, and personally manageable are often viewed as less threatening than those associated with external actors or more complex supply chains in which consumers have limited visibility or influence over safety practices (Mucinhato et al. 2026). This could explain why self‐prepared food retained stronger confidence, as personal involvement from the participants likely increases both perceived transparency and trust. Overall, the pattern suggests that confidence in food supply chain actors is influenced by perceptions of personal competence, prior knowledge, transparency, and familiarity with food system operations (Macready et al. 2020; IFIC 2025). Beyond shifts in confidence, these findings suggest that the documentary also shaped how young adult participants perceived the broader importance of food safety, with emotional engagement in the documentary narrative contributing to this effect.

### 3.3 Impact of Documentary Narrative on Educational Value of Food Safety Communication

#### 3.3.1 Perceived Importance of Food Safety Changes Over Time

Watching the documentary provoked emotional responses and diminished viewers’ confidence in the presented food supply chain actors, and the study further examined whether these reactions influenced viewers' perceived importance of food safety. Survey results from nonfood science participants revealed a statistically significant change between pre‐ and post‐surveys, from *M* = 4.237 (SD = 0.817; Md = 4) to *M* = 4.576 (SD = 0.593; Md = 5) (*r* = 0.529; *p *< 0.001), suggesting that the documentary exposure shifted participants' short‐term perceptions of the importance of food safety. In contrast, food science participants showed no statistically significant change from pre‐ to post‐surveys. Post‐to‐follow‐up comparisons were also not statistically significant in either study (Table 4). This indicates that while the documentary had an immediate effect on nonfood science participants, the effect was not sustained over time and was not clearly observed among students in food science majors. This may suggest a temporary attitudinal shift, rather than a durable change in young adult viewers' perceived importance of food safety.

**TABLE 4:** Self‐reported importance of food safety before and after documentary intervention on a 5‐point Likert scale (1 = *not at all important*, 5 = *very important*).

	Pre survey		Post survey		Wilcoxon *Z*	*p*	*r*
	*M* ± SD	Md	*M* ± SD	Md			
Study 1 (*n* = 19)							
Importance of food safety	4.737 ± 0.452	5	4.842 ± 0.375	5	1.414		0.324
Study 2 (*n* = 59)							
Importance of food safety	4.237 ± 0.817	4	4.576 ± 0.593	5	4.066	***	0.529

	Post survey		Follow‐up survey		Wilcoxon *Z*	*p*	*r*
	*M* ± SD	Md	*M* ± SD	Md			
Study 1 (*n* = 19)							
Importance of Food Safety	4.842 ± 0.375	5	4.789 ± 0.535	5	−0.447		0.103
Study 2 (*n* = 59)							
Importance of Food Safety	4.576 ± 0.593	5	4.508 ± 0.653	5	−1.069		0.139

Abbreviations: *M* ± SD, mean ± standard deviation; Md, median; *p*, *p*‐value; *r*, effect size.

***p < 0.001.

Food science students consistently assigned greater importance to food safety than their nonfood science peers. This difference may reflect stronger domain‐specific knowledge and greater familiarity with food safety concepts acquired through formal academic training while obtaining their degree. Existing research among university students suggests that formal education in food‐ and health‐related disciplines can shape domain knowledge, attitudes, and interpretation of food‐related information (Jin and Han 2019; Deeb et al. 2024). Jin and Han (2019) demonstrated that food safety education can shape students’ knowledge, trust, and risk perception, affecting how individuals evaluate controversial food information. Accordingly, participants with stronger prior knowledge of the documentary topic may have been less susceptible to short‐term emotional or perceptual shifts following documentary exposure, which may explain the limited change observed among food science participants.

Although only nonfood science participants in Study 2 showed a statistically significant immediate increase, both studies followed a similar pattern: perceived importance of food safety increased immediately after documentary viewing, then declined slightly by the 9‐day follow‐up. While the attenuation suggests that the documentary's influence weakened over time, follow‐up scores remained higher than baseline levels (pre‐survey), indicating that documentary exposure may have left a modest residual impression. However, because these longer‐term differences were not statistically significant, this possible sustained effect should be interpreted cautiously. The relatively elevated mean scores observed across both studies (Table 4) further indicate that food safety was already perceived as a personally relevant issue among young adult participants.

The pattern is consistent with previous research indicating that documentary films can influence audience attitudes and perceptions regarding socially relevant issues, including environmental sustainability, food and health topics, and animal welfare (Cottone and Byrd‐Bredbenner 2007; Dissen and Crowell 2020; Waller and Iluzada 2020). The decline in scores observed at the follow‐up survey suggests that the effect of documentary exposure may diminish over time in the absence of reinforcement or continued engagement with the topic. Similar findings were reported by Majestya and Irwanto (2025), who found that environmental documentaries were more effective at producing immediate perceptual shifts than long‐term changes in values or beliefs. Even if the residual impression beyond the immediate viewing appears modest, it may be meaningful in the context of food safety communication, in which even a small increase in perceived importance could encourage greater attention to food safety risk information or safer food‐handling practices.

#### 3.3.2 Storytelling as a Catalyst for Food Safety Reflection

Participants from both studies generally described the documentary as engaging and accessible. Focus group discussion findings provided additional insight into the narrative features that contributed to its perceived educational value. Participants emphasized that personal narratives and real‐world outbreak examples transformed foodborne illness from an abstract concept that “only happens to others” into a relatable risk that could affect one's own family or community. As one participant explained, “*Watching it, […] kind of does become more personal that way. It's like this could happen to anybody. It could have happened to me or my family*” (FG10 P55 S2). Nonfood science students described *Poisoned* as “*a good intro to food safety*” (FG8 P47 S2) and an engaging way to learn about food safety: “*Yeah, it's real‐life based learning which you can, like, actually understand all the things which are happening around you, and you can feel more connected that way*” (FG7 P34 S2). Many reported during focus group discussions that the film inspired reflection and motivated them to seek further information about food safety due to its accessibility through simple scientific knowledge: “*Documentary is definitely like a great tool to kind of intrigue people, like interest into the thing they're talking about, because it doesn't contain a lot of, like, scientific data, it's really easy for students to understand*” (FG3 P16 S2).

However, participants also identified limitations in the documentary's ability to provide a comprehensive understanding of the US food safety system. Some characterized the documentary as an “incomplete essay,” suggesting that it underexplains or omits key details and risks misinterpretation. Several felt that the documentary failed to present a balanced view of the factors influencing food safety, such as working conditions or regulatory complexity. As one participant commented, “*[…] I think they also left out a lot of the variables. It's very easy to be like, oh yeah, the workers aren't doing enough. I feel like they could have touched on that a little bit*” (FG2 P14 S2). Many participants agreed that the documentary was an effective entry point; however, it provided insufficient practical guidance to deepen viewers’ understanding and promote behavioral change. “*I think it's a good starting point. One thing it kind of leaves out is, like, probably safer ways to do a lot of things, like it doesn't talk about. […] how to clean your meats or not clean your meats, or how to properly wash the lettuce. […]*” (FG3 P15 S2).

These responses suggest that storytelling allowed participants to engage with food safety by making the issue personally relevant and emotionally salient. This interpretation is consistent with Sundin et al. (2018), who suggest that storytelling can strengthen audience engagement with complex scientific communication by making messages more relatable and action‐oriented. However, the findings also reveal a tension between accessibility and scientific depth. Participants valued the documentary because of its accessible content that “*doesn't contain a lot of, like, scientific data*,” making the topic easier to understand, yet they described it as incomplete for the omission of contextual information and practical guidance. This apparent contradiction suggests that reducing scientific complexity may lower barriers to engagement, while also limiting the depth of understanding needed to interpret complex food safety issues accurately. This tension reflects Biltekoff's (2024) critique of food communication approaches that assume providing more scientific knowledge is sufficient to address public concerns. She suggests that individual interpretation of food‐related information is more complex and must be considered alongside individuals’ values, assumptions, and questions of scientific authority within the food system. Consistent with this, Howell et al. (2025) found that engagement with science documentaries varied depending on topic interest and emotional framing. In their study, lower engagement was associated with a documentary with scientific content presented through a scientist describing another person's research rather than personally relatable or emotionally compelling narratives.

The emotional response may also be understood through transformative learning theory, in which emotionally charged experiences can stimulate critical reflection by disrupting existing assumptions and prompting individuals to reconsider their beliefs and behaviors (Smith 2014). In this study, participants reported reflecting on their own food‐handling habits after watching the documentary, suggesting that emotional storytelling may have contributed to this type of reflective learning. Nevertheless, emotional engagement and increased awareness do not necessarily provide viewers with the knowledge needed to make evidence‐based food safety decisions. Participants’ requests for clearer practical guidance indicate that narrative engagement alone may not be sufficient for sustained behavior change. Although storytelling can capture audience attention and foster emotional connection among viewers (Jialei 2024), narrative‐driven communication may also selectively frame or oversimplify nuanced information (Makarova‐Krentsel 2025). Documentary films using storytelling may therefore serve as a valuable entry point for food safety reflection by making complex issues more relatable and personally relevant to viewers regardless of their prior knowledge or background in the topic. Food safety educators and industry stakeholders should consider that audiences may use documentaries such as *Poisoned* as a source of food safety information even when they are not intended as formal educational tools.

#### 3.3.3 From Impact to Insight: Addressing Bias and Limits of Using a Documentary as a Potential Educational Resource

Participants from both food science and nonfood science majors generally viewed documentaries as valuable educational resources, but concerns emerged regarding perceived bias in their production. Several participants reported that the filming and editing techniques appeared to be designed to provoke strong emotional responses, such as fear or frustration, while presenting the content as factual. This emotional framing was viewed as potentially detrimental to learning, as emotional responses were believed to interfere with the viewer's critical thinking and information processing. One participant from *Study 2* commented, “*Yeah, I definitely say watch out for bias. Like, when they're, like, interviewing the two government officials that kind of cut out everything they're saying, except for, like, the what they want to be shown*” (FG7 P36 S2). Such practices led some participants to question the credibility and the objectivity of the information presented. These findings align with existing research, emphasizing the importance of framing in documentary viewing. As Smith (2014) shows, documentary features and form, including stylistic conventions, can evoke emotional responses that shape viewers’ interpretation and influence how they understand the presented issues. Although documentaries are often presented as a window into reality, filmmakers carefully construct narratives shaped by artistic and communicative choices. The selection and framing of documentary subject matter often reflect both audience interest and filmmakers’ intent to convey a specific message. Documentary films are often meant not only to inform but also to direct audience attention toward a particular issue, thereby potentially increasing the perceived importance of specific issues. In *Poisoned: The Dirty Truth About Your Food*, this framing centers on systemic food safety failures in the US food production and regulatory system, particularly regarding foodborne pathogen contamination risks. Corner (1996) explains that documentary style frequently assumes transparency while masking its mediated nature, often using reflexive narration to invite critical engagement from viewers. This highlights that documentary narratives are partial and positioned rather than neutral representations of reality (Danish 2025). Prior research similarly demonstrates that a documentary can influence short‐term food‐related attitudes and behavioral intentions. For example, exposure to food‐related documentaries has been shown to reduce consumption of certain products, including meat and other animal products (Mathur et al. 2021). *Food, Inc*., a 2008 documentary that critically examines the US industrial food system, highlighted concerns related to food safety, animal welfare, and environmental sustainability. Ma et al. (2020) reported that after *Food, Inc*. was released, it was associated with an increase in consumption of some organic foods, such as yogurt; however, these effects diminished over time (Ma et al. 2020). The findings from the current study reinforce this literature by suggesting that documentary‐influenced behavioral intentions are not necessarily aligned with evidence‐based food safety recommendations. Another study analyzing the *Food, Inc*. discussion guide, developed by the Center for Eco Literacy, shows how food‐related concerns can be framed within a broader system of social, economic, regulatory, and production factors rather than simply the responsibility of consumers alone (Biltekoff 2016). Biltekoff's study of the guide detailed that it encouraged consideration of who is responsible for food‐related issues and the roles of institutions, public health, and the food system. This broader framing of responsibility is relevant to the present findings, as participants’ responses reflected both concerns about food supply chain actors and consideration of changes in their own food handling practices.

Despite these broader concerns, participants in both studies reported during focus group discussion that they intended to make some changes in their choice of foods after viewing outbreak‐related content in the documentary, often reflecting heightened or exaggerated risk responses. Several participants described their willingness to avoid certain food products, particularly romaine or bagged lettuce: “*I think definitely like the romaine lettuce, that officials are just like not eating it, like they're steering clear of romaine lettuce, so I'll stay away from bagged lettuce as well*” (FG1 P2 S1). The documentary also appeared to reinforce or create unsafe food‐handling practices, notably washing raw poultry, “*[…] I even sometimes wash my chicken. Yeah, I do. That's the only thing I can think of that I have to do, which I probably could be doing more*” (FG6 P33 S2). Participants reported their intention to wash raw poultry in their kitchen sinks following documentary viewing. However, this practice poses significant food safety risks, as it increases the risk of foodborne illness by cross‐contamination in the kitchen, without effectively removing pathogens, such as *Salmonella* or *Campylobacter* (Carmody et al. 2022; CDC 2024; USDA FSIS 2024). Consumer communication campaigns emphasized the potential risks and that proper cooking is the best practice to eliminate harmful bacteria (Henley et al. 2016). Research shows that many consumers who wash poultry are unaware of the potential risk and that exposure to clear explanatory messaging significantly increased confidence in adopting safe handling practices (Vatral et al. 2022). Within this context, the present findings suggest that the documentary *Poisoned* may unwillingly reinforce perceptions and behavioral intentions that are inconsistent with established food safety guidelines. When coupled with viewers expressed distrust in food regulators, these findings raise broader concerns regarding the use of emotionally framed documentary narratives for educational purposes.

Participants also reported their intentions to share information they learned from the documentary with others, particularly family members and friends. As the study focused on young adults, social media emerged as an important platform. This willingness to share what they learned was illustrated when, shortly after data collection, one nonfood science participant posted on social media a picture of a campus sandwich restaurant. The post included the caption, “*me freaking out because they use bagged lettuce in my sandwich*,” followed by “*iykyk*,” meaning “*If you know, you know*.” This example demonstrates the power of social media platforms for this generation to learn from others; however, it also reflects the very rapid, easy spread of misinformation. Social media platforms facilitate peer learning and engagement and could be a great tool for food safety communication to younger populations (Sivakumar et al. 2023). However, they also enable the rapid dissemination of information that may lack scientific context or accuracy, increasing the risk of misinterpretation and misinformation (Rodrigues et al. 2024). Documentaries may increase food safety awareness and stimulate information sharing, but emotionally framed narratives also may promote misinformation, distrust, and behavioral intentions that are inconsistent with evidence‐based food safety recommendations. These findings highlight the importance of balanced food safety communication. Such communication should present both the strengths and failures of the food system, explain the factors underlying actors’ decisions and practices, and provide audiences with sufficient context to understand food safety issues from multiple perspectives.

## 4 Limitations

Although the study has been carefully designed and implemented, several limitations should still be considered. First, the use of a convenience sample consisting of undergraduate students from one Indiana university may introduce a potential bias to the study. This limits the representativeness of the participants and may restrict the transferability of the findings beyond similar populations. The relatively small sample size also may have been a limitation of this study. However, this limitation should be considered within the context of the mixed‐methods design. In the qualitative component, the strength of the findings depends not only on the sample size but also on the information richness provided by the participants and the rigor of the analytic process (Krueger and Casey 2015). Participants across both studies represented a relatively focused population with a shared intervention experience of viewing the documentary. The homogeneity allowed the study to explore participants’ perceptions in depth within a specific context despite the limited sample size. Study findings should be considered a starting point, and further research with larger, more diverse populations is needed to determine if these findings are transferable to broader populations. Participants in both studies lived on the same university campus and completed the follow‐up assessment within 9 days of viewing the documentary, which may have limited major differences in their general information environment. However, food science students may have encountered additional food safety‐related information through their coursework, whereas nonfood science majors may not have had comparable exposure. Since exposure to other food safety information was not measured, its potential influence on follow‐up responses or differences observed between the two studies cannot be determined. Further research could account for exposure to food safety information during the follow‐up period and incorporate repeated assessments to measure longer periods. Additionally, the study was not designed to determine whether participants’ emotional engagement translated into sustained changes in food safety behavior. Future studies could include repeated follow‐up assessments and direct or clearly defined self‐reported behavioral measures to examine whether emotional engagement leads to sustained changes in food safety practices. Second, focus groups are valuable qualitative methods that allow researchers to explore participants' opinions on a specific topic, often yielding rich, nuanced data from group discussion. They provide deeper insight into the reasons behind participants' perceptions and behaviors. Focus group methodology also presents challenges. Participants may conform to the opinions of the group (Krueger and Casey 2015), with dominant participants potentially influencing the discussion dynamic (Then et al. 2014). Even if moderators were trained to facilitate discussion and minimize these effects, such influence cannot be entirely eliminated. Despite these limitations, integrating qualitative and quantitative approaches strengthens the study by providing deeper insights (Clark and Plano Clark 2022).

## 5 Conclusion

These two studies reinforce previous evidence that documentary films that use emotionally driven storytelling, while highly effective at capturing attention and fostering personal connection, are a double‐edged sword in the context of food safety. While the emotional narratives transform abstract risks into relatable experiences, the analyzed documentary increase viewers’ awareness and personal relevance, and in some cases, motivate them to adopt safer food practices. However, the findings also indicate increased negative emotional reactions, reduced confidence in food supply chain actors, and occasional misinterpretations of safe food handling practices. These effects were particularly pronounced among students enrolled in majors other than food science. Rather than positioning *Poisoned* as an educational tool available to a large audience, this study highlights it as an important lesson for the food industry, government agencies, educators, and media creators. The strong reactions generated by the documentary, and the observed decline in confidence in food supply chain actors, suggest a need for more proactive, transparent, and collaborative communication about how the food system operates, how food safety risks are managed, and what consumers can do to make informed decisions. Although documentaries can raise awareness and engage audiences, their educational value may be strengthened through balanced food safety communication. Such communication should present both the strengths and failures of the food system, explain the factors underlying actors’ decisions and practices, and provide sufficient context for viewers to understand the issue from multiple perspectives. Food industry and government communicators could benefit from working more closely with communication professionals, educators, and media creators to provide clear, transparent, and evidence‐based information that contextualizes food safety risks and explains the roles of different actors within the US food supply chain. Integrating emotionally engaging content with structured, evidence‐based instruction could help balance affective responses with accurate understanding, ultimately supporting more informed perceptions and sustained behavioral change in food safety contexts. In an educational setting, documentary‐based materials through the example of *Poisoned* could be paired with guided discussion, fact‐checking activities, and evidence‐based resources that clarify misunderstood food safety practices and provide practical guidance requested by viewers. These findings should be interpreted within the limitations of the present work, particularly considering the small convenience samples recruited from a single university, which limits the generalizability beyond similar populations. The observed patterns suggest several practical implications. Future research should examine these patterns using larger and more diverse samples, while incorporating baseline assessments of food safety knowledge and longer‐term measures of perceptions, trust, and behavioral outcomes following documentary exposure beyond 9 days.

## Author Contributions

**Fanny Gozzi**: conceptualization, methodology, investigation, validation, visualization, writing – review and editing, writing – original draft. **Shams Adigozalzade**: investigation, visualization, writing – original draft. **Yaohua Feng**: supervision, funding acquisition, validation, writing – review and editing.

## Conflicts of Interest

The authors declare no conflicts of interest.

## Supporting information




**Supporting File 1**: jfds71589‐sup‐0001‐SuppMat.docx

**Supporting File2**: jfds71589‐sup‐0001‐SuppMat.docx

**Supporting File 3**: jfds71589‐sup‐0001‐SuppMat.docx

## References

[jfds71589-bib-0001] Bánáti, D. , and A. Baicu . 2024. “Food Legislation and the Consumers: The EU Perspective.” In Consumer Perception of Food Safety in Europe, edited by Diana Bogueva . Springer.

[jfds71589-bib-0002] Berejena, T. , and F. Malongane . 2025. “The Impact of the Food Safety Community Based Education Programme (FSCBEP) on the Food Safety Knowledge (FSK) Level of Johannesburg's Peri‐Urban Dwellers.” International Journal of Community Well‐Being 8, no. 3: 761–778. 10.1007/s42413-025-00266-z.

[jfds71589-bib-0003] Berglund, Z. , S. Simsek , and Y. Feng . 2024. “Effectiveness of Online Food‐Safety Educational Programs: A Systematic Review, Random‐Effects Meta‐Analysis, and Thematic Synthesis.” Foods 13, no. 5: 794. 10.3390/foods13050794. PMC10931379 38472907

[jfds71589-bib-0004] Bieniek‐Tobasco, A. , S. McCormick , R. N. Rimal , C. B. Harrington , M. Shafer , and H. Shaikh . 2019. “Communicating Climate Change Through Documentary Film: Imagery, Emotion, and Efficacy.” Climatic Change 154: 1–18. 10.1007/s10584-019-02408-7.

[jfds71589-bib-0005] Biltekoff, C. 2016. “The Politics of Food Anti‐Politics.” Gastronomica 16, no. 4: 44–57. 10.1525/gfc.2016.16.4.44.

[jfds71589-bib-0006] Biltekoff, C. 2024. Real Food, Real Facts: Processed Food and the Politics of Knowledge. University of California Press. https://www.ucpress.edu/books/real‐food‐real‐facts/paper.

[jfds71589-bib-0007] Boissat, L. , L. Thomas‐Walters , and D. Veríssimo . 2021. “Nature Documentaries as Catalysts for Change: Mapping Out the ‘Blackfish Effect’.” People and Nature 3, no. 6: 1179–1192. 10.1002/pan3.10221.

[jfds71589-bib-0008] Bondebjerg, I. b. 2014. “Documentary and Cognitive Theory: Narrative, Emotion and Memory.” Media and Communication 2, no. 1: 13–22. 10.17645/mac.v2i1.17.

[jfds71589-bib-0009] Braun, V. , and V. Clarke . 2006. “Using Thematic Analysis in Psychology.” Qualitative Research in Psychology 3, no. 2: 77–101. 10.1191/1478088706qp063oa.

[jfds71589-bib-0010] Bruhn, C. , and Y. Feng . 2021. “Exploring Consumer Response to Labeling a Processing Aid That Enhances Food Safety.” Food Protection Trends 41, no. 3: 305–313. https://www.foodprotection.org/members/fpt‐archive‐articles/2021‐05‐exploring‐consumer‐response‐to‐labeling‐a‐processing‐aid‐that‐enhances‐food‐safety/.

[jfds71589-bib-0011] Carmody, C. D. , R. C. Mueller , B. M. Grodner , O. Chlumsky , J. N. Wilking , and S. G. McCalla . 2022. “Chickensplash! Exploring the Health Concerns of Washing Raw Chicken.” Physics of Fluids 34, no. 3: 031910. 10.1063/5.0083979. PMC8958727 35392485

[jfds71589-bib-0012] Centers for Disease Control and Prevention . 2024. Chicken and Food Poisoning. Food Safety. Accessed December 19, 2025. https://www.cdc.gov/food‐safety/foods/chicken.html.

[jfds71589-bib-0013] Centers for Disease Control and Prevention . 2025. Facts About Food Poisoning. Food Safety. Accessed November 24, 2025. https://www.cdc.gov/food‐safety/data‐research/facts‐stats/index.html.

[jfds71589-bib-0014] Chen, H. , J. C. Archila‐Godínez , L. Klinestiver , et al. 2023. “Implementation of a Dialogue‐Based Food Safety Education Program for Low‐Income Families.” Food Control 143: 109293. 10.1016/j.foodcont.2022.109293.

[jfds71589-bib-0015] Chen, K. , Z. Lin , I. do Prado , Z. Mohammad , K. M. Acosta , and S. A. Sirsat . 2024. “College Students' Domestic Kitchen Food Safety Perceptions and Knowledge.” Journal of Extension 61, no. 4: 12. 10.34068/joe.61.04.12.

[jfds71589-bib-0016] Chuang, E. , M. Thomas , and Y. Feng . 2021. “Young Adult Food Safety Knowledge Gaps and Perceptions of Roommates' Food Handling Practices: A Survey of University Students in Indiana.” Food Control 126: 108055. 10.1016/j.foodcont.2021.108055.

[jfds71589-bib-0017] Clark, R. S. , and V. L. Plano Clark . 2022. “The Use of Mixed Methods to Advance Positive Psychology: A Methodological Review.” International Journal of Wellbeing 12, no. 3: 35–55. 10.5502/ijw.v12i3.2017.

[jfds71589-bib-0018] Cohen, J. 1988. Statistical Power Analysis for the Behavioral Sciences. 2nd ed. Lawrence Erlbaum Associates, 77–81.

[jfds71589-bib-0019] Corner, J. 1996. The Art of Record: A Critical Introduction. Manchester University Press.

[jfds71589-bib-0020] Cottone, E. , and C. Byrd‐Bredbenner . 2007. “Knowledge and Psychosocial Effects of the Film *Super Size Me* on Young Adults.” Journal of the American Dietetic Association 107, no. 7: 1197–1203. 10.1016/j.jada.2007.04.005. 17604751

[jfds71589-bib-0021] Danish, I. 2025. “The Ethics of Documentary Filmmaking: Truth vs. Narrative.” Sociology & Cultural Research Review 4, no. 1: 1020–1029. https://www.scrrjournal.com/index.php/14/article/view/379.

[jfds71589-bib-0022] Daugelat, M.‐C. , G. Bettina , A. S. Helen , S. Kathrin , K. Joachim , and G. K. Elisabeth . 2024. “Participatory Development of Evidence‐Based Patient Narrative Videos for Patients With Eating Disorders: A Methodological Approach and Pilot Data.” Journal of Eating Disorders 12, no. 188: 00. 10.1186/s40337-024-01146-1. PMC11580210 39567996

[jfds71589-bib-0023] Deeb, N. , F. Naja , L. Nasreddine , S. Kharroubi , N. Darwiche , and N. Hwalla . 2024. “Nutrition Knowledge, Attitudes, and Lifestyle Practices That May Lead to Breast Cancer Risk Reduction Among Female University Students in Lebanon.” Nutrients 16, no. 7: 1095. 10.3390/nu16071095. PMC11013092 38613128

[jfds71589-bib-0024] Dilley, A. , B. R. McFadde , J. L. Mitchell , and J. M. Thompson . 2026. “Claiming Confidence: U.S. Consumer Trust in Meat and Poultry Claims.” Journal of Animal Science 104: skag032. 10.1093/jas/skag032. PMC12971111 41678672

[jfds71589-bib-0025] Dissen, A. , and T. Crowell . 2020. “We Are What We Eat: Assessing the Use of a Documentary Film as an Educational Tool to Change Students' Nutritional Attitudes and Behaviors.” American Journal of Lifestyle Medicine 16, no. 2: 241–250. 10.1177/1559827620903701. PMC8971699 35370516

[jfds71589-bib-0026] Feng, Y. , and C. M. Bruhn . 2025. “Consumer Acceptance of Nonthermal Food Processing Technologies.” In Nonthermal Processing Technologies for Food, edited by Howard Q. Zhang , V. M. Balasubramaniam , C. Patrick Dunne , James T. C. Yuan , Daniel F. Farkas , and Gustavo V. Barbosa‐Cánovas . John Wiley & Sons. 10.1002/9781119265665.ch39.

[jfds71589-bib-0027] Gugenishvili, I. , and A. Laine‐Kronberg . 2025. “The Influence of Prior Knowledge and Perceived Impact on the Connection Between Attitudes and Sustainable Behavior.” Sustainable Development 33: 4099–4111. 10.1002/sd.3335.

[jfds71589-bib-0028] Henley, S. C. 2025. “Let's Talk About Food Safety Awareness and Education—Food Safety Scientists Weigh in on Netflix's Documentary *Poisoned* .” Food Protection Trends 45, no. 4: 282–283.

[jfds71589-bib-0029] Henley, S. C. , J. Gleason , and J. J. Quinlan . 2016. “Don't Wash Your Chicken! A Food Safety Education Campaign to Address a Common Food Mishandling Practice.” Food Protection Trends 36, no. 1: 43–53.

[jfds71589-bib-0030] Howell, E. L. , S. L. Behrman , E. Kirschner , and S. S. Goodwin . 2025. “Storytelling in Science Film: Narrative Engagement Relates to Greater Knowledge, Interest, and Identification With Science.” Science Communication 47, no. 2: 211–249. 10.1177/10755470241269885.

[jfds71589-bib-0031] International Food Information Council (IFIC) . 2020. “Public Comments to FSIS Meeting, Food Safety: Consumer Outreach and Education Today and for the Future.” Accessed January 27, 2026. https://ific.org/wp‐content/uploads/2025/04/IFIC‐Public‐Comments‐to‐FSIS.Consumer‐Outreach‐and‐Education.pdf.

[jfds71589-bib-0032] International Food Information Council (IFIC) . 2025. “2025 IFIC Food & Health Survey: The Full Report.” Accessed December 19, 2025. https://ific.org/research/2025‐food‐health‐survey.

[jfds71589-bib-0033] International Food Information Council (IFIC) . 2025. “A Focus on Food & Ingredient Safety.” Accessed January 27, 2026. https://ific.org/wp‐content/uploads/2025/07/2025‐IFIC‐Food‐Health‐Survey‐Food‐Ingredient‐Safety.pdf.

[jfds71589-bib-0034] Ivankova, N. V. , J. W. Creswell , and S. L. Stick . 2006. “Using Mixed‐Methods Sequential Explanatory Design: From Theory to Practice.” Field Methods 18, no. 1: 3–20. 10.1177/1525822x05282260.

[jfds71589-bib-0035] Jaeger, H. , D. Knorr , E. Szabó , J. Hámori , and D. Bánáti . 2015. “Impact of Terminology on Consumer Acceptance of Emerging Technologies Through the Example of PEF Technology.” Innovative Food Science & Emerging Technologies 29: 87–93. 10.1016/j.ifset.2014.12.004.

[jfds71589-bib-0036] Jialei, J. 2024. “Emotions Are What Will Draw People in: A Study of Critical Affective Literacy Through Digital Storytelling.” Journal of Adolescent & Adult Literacy 67: 253–263. 10.1002/jaal.1322.

[jfds71589-bib-0037] Jin, H. J. , and D.‐H. Han . 2019. “College Students' Experience of a Food Safety Class and Their Responses to the MSG Issue.” International Journal of Environmental Research and Public Health 16, no. 16: 2977. 10.3390/ijerph16162977. PMC6719175 31430891

[jfds71589-bib-0038] Kankal, B. , S. Kumar Patra , and R. Panda . 2023. “Pedagogy Innovation and Integration of Films in Management Education: Review and Research Paradigms.” International Journal of Management Education 21, no. 2: 100804. 10.1016/j.ijme.2023.100804.

[jfds71589-bib-0039] Konijn, E. A. , J. H. v. d. Molen , and S. v. Nes . 2009. “Emotions Bias Perceptions of Realism in Audiovisual Media: Why We May Take Fiction for Real.” Discourse Processes 46, no. 4: 309–340. 10.1080/01638530902728546.

[jfds71589-bib-0040] Kramer, A. E , E. R. Hoover , N. Hedeen , et al. 2023. “Development of an Empirically Derived Measure of Food Safety Culture in Restaurants.” Journal of Food Protection 86, no. 3: 100043. 10.1016/j.jfp.2023.100043. PMC10018426 36916550

[jfds71589-bib-0041] Krueger, R. A. , and M. A. Casey . 2015. Focus Groups: A Practical Guide for Applied Research. 5th ed. Sage Publications.

[jfds71589-bib-0042] Lin, S.‐J. 2013. “Perceived Impact of a Documentary Film: An Investigation of the First‐Person Effect and Its Implications for Environmental Issues.” Science Communication 35, no. 6: 708–733. 10.1177/1075547013478204.

[jfds71589-bib-0043] Ma, J. , S. Seenivasan , and B. Yan . 2020. “Media Influences on Consumption Trends: Effects of the Film *Food, Inc*. on Organic Food Sales in the U.S.” International Journal of Research in Marketing 37, no. 2: 320–335. 10.1016/j.ijresmar.2019.08.004.

[jfds71589-bib-0044] Macready, A. L. , S. Hieke , M. Klimczuk‐Kochańska , S. Szumiał , L. Vranken , and K. G. Grunert . 2020. “Consumer Trust in the Food Value Chain and its Impact on Consumer Confidence: A Model for Assessing Consumer Trust and Evidence From a 5‐Country Study in Europe.” Food Policy 92: 101880. 10.1016/j.foodpol.2020.101880.

[jfds71589-bib-0045] Majestya, N. , and I. Irwanto . 2025. “Beyond Behavioral Change: Evaluating the Impact of Environmental Films on Audience Perceptions and Beliefs About Food System Issues.” Frontiers in Communication 10: 1519348. 10.3389/fcomm.2025.1519348.

[jfds71589-bib-0046] Makarova‐Krentsel, I. 2025. “Eyewitness Testimony in Documentary Film: A Study of Trust, Memory, and the Limitations of Personal Narrative.” Universal Library of Arts and Humanities 2, no. 3: 83–91. 10.70315/uloap.ulahu.2025.0203012.

[jfds71589-bib-0047] Marklinder, I. , G. Eskhult , R. Ahlgren , et al. 2022. “A Structural Equation Model Demonstrating the Relationship Between Food Safety Background, Knowledge, Attitudes and Behaviour Among Swedish Students.” Foods 11, no. 11: 1595. 10.3390/foods11111595. PMC9180796 35681344

[jfds71589-bib-0048] Mathur, M. B. , J. R. Peacook , T. N. Robinson , and C. D. Gardner . 2021. “Effectiveness of a Theory‐Informed Documentary to Reduce Consumption of Meat and Animal Products: Three Randomized Controlled Experiments.” Nutrients 13, no. 12: 4555. 10.3390/nu13124555. PMC8708224 34960107

[jfds71589-bib-0049] McKay, C. , J. Pate , T. Mirabito , and E. Anaza . 2022. “Engaging With Wheelchair Basketball: Analyzing Viewer Attitudes and Actions Toward *The Rebound: A Wheelchair Basketball Story* (documentary).” Sport in Society 25, no. 5: 963–981. 10.1080/17430437.2020.1816964.

[jfds71589-bib-0050] Morse, J. M. 1991. “Evaluating Qualitative Research.” Qualitative Health Research 1, no. 3: 283–286. 10.1177/104973239100100301.

[jfds71589-bib-0051] Mosier, S. L. 2023. “An Evaluation of the Role of US Consumer's Institutional Trust for Food Eco‐Label Preferences.” World Food Policy 9, no. 1: 50–71. 10.1002/wfp2.12054.

[jfds71589-bib-0052] Mubangizi, V. , J. Plastow , F. Nakaggwa , et al. 2022. “Assessing Changes in Knowledge, Attitudes, and Intentions to Use Family Planning After Watching Documentary and Drama Health Education Films: A Qualitative Study.” Reproductive Health 19, no. 65: 00. 10.1186/s12978-022-01370-5. PMC8917732 35279189

[jfds71589-bib-0053] Mucinhato, R. , D. Moreira , L. M. Zanin , et al. 2026. “Beyond Knowledge: Perceived Behavioral Control and Risk Perception Mediate Consumer's Food Safety Behavior in South America.” Food Research International 225: 118018. 10.1016/j.foodres.2025.118018. 41508444

[jfds71589-bib-0054] Onwuegbuzie, A. J. , W. B. Dickinson , N. L. Leech , and A. G. Zoran . 2009. “A Qualitative Framework for Collecting and Analyzing Data in Focus Group Research.” International Journal of Qualitative Methods 8, no. 3: 1–21. 10.1177/160940690900800301.

[jfds71589-bib-0055] Or, S. , M. Naama , R. Doron , L. Oren , T. Yariv , and T.‐O. Nurit . 2023. “The Impact of Testimony Journalism on Audience Engagement: An Experimental Investigation of the Effects of Point of View.” Journalism Studies 24, no. 5: 1–21. 10.1080/1461670x.2023.2173957.

[jfds71589-bib-0056] Rodrigues, F. , R. Newell , B. Giridhara Rathnaiah , T. Chatterjee , S. Nimrat Kaur , and L. Gupta . 2024. “The Social Media Infodemic of Health‐Related Misinformation and Technical Solutions.” Health Policy and Technology 13, no. 2: 100846. 10.1016/j.hlpt.2024.100846.

[jfds71589-bib-0057] Shabiralyani, G. , S. Hasan Khuram , N. Hamad , and N. Iqbal . 2015. “Impact of Visual Aids in Enhancing the Learning Process Case Research.” Journal of Education and Practice 6: 227–233. https://eric.ed.gov/?id=EJ1079541.

[jfds71589-bib-0058] Shaffer, V. A. , E. S. Focella , A. Hathaway , L. D. Scherer , and B. J. Zikmund‐Fisher . 2018. “On the Usefulness of Narratives: An Interdisciplinary Review and Theoretical Model.” Annals of Behavioral Medicine 52, no. 5: 429–442. 10.1093/abm/kax008. PMC6369912 29684135

[jfds71589-bib-0059] Sivakumar, A. , J. Sudarsan , and S. Shahenaz . 2023. “Social Media Influence on Students' Knowledge Sharing and Learning: An Empirical Study.” Education Sciences 13, no. 7: 745. 10.3390/educsci13070745.

[jfds71589-bib-0060] Smith, H. J. 2014. “Emotional Responses to Documentary Viewing and the Potential for Transformative Teaching.” Teaching Education 25, no. 2: 217–238. 10.1080/10476210.2012.762351.

[jfds71589-bib-0061] Soechtig, S. 2023. Poisoned: The Dirty Truth About Your Food. Netflix. https://www.netflix.com/title/81460481.

[jfds71589-bib-0062] Statista . 2021. “Share of Adults With a Netflix Subscription in the United States as of August 2021, by Age Group.” Statista Research Department. Accessed August 19, 2026. https://www.statista.com/statistics/742108/netflix‐subscription‐adults‐usa‐by‐age/.

[jfds71589-bib-0063] Sundin, A. , K. Andersson , and R. Watt . 2018. “Rethinking Communication: Integrating Storytelling for Increased Stakeholder Engagement in Environmental Evidence Synthesis.” Environmental Evidence 7: 6. 10.1186/s13750-018-0116-4.

[jfds71589-bib-0064] Taber, K. S. 2018. “The Use of Cronbach's Alpha When Developing and Reporting Research Instruments in Science Education.” Research in Science Education 48: 1273–1296.

[jfds71589-bib-0065] Tam, R. , B. Yassa , H. Parker , H. O'Connor , and M. Allman‐Farinelli . 2017. “University Students' On‐Campus Food Purchasing Behaviors, Preferences, and Opinions on Food Availability.” Nutrition 37: 7–13. 10.1016/j.nut.2016.07.007. 28359366

[jfds71589-bib-0066] Then, K. L. , J. A. Rankin , and E. Ali . 2014. “Focus Group Research: What Is It and How Can It be Used?” Canadian Journal of Cardiovascular Nursing 24, no. 1: 16–22. 24660275

[jfds71589-bib-0067] U.S. Department of Agriculture, Food and Nutrition Administration (USDA FNA) . 2025. “Videos: Food Safety Flashes.” Accessed February 10, 2026. https://www.fns.usda.gov/fs/food‐safety‐flashes.

[jfds71589-bib-0068] U.S. Department of Agriculture, Food Safety and Inspection Service (USDA FSIS) . 2024. “Washing Food: Does It Promote Food Safety?.” Accessed December 19, 2025. https://www.fsis.usda.gov/food‐safety/safe‐food‐handling‐and‐preparation/food‐safety‐basics/washing‐food‐does‐it‐promote‐food.

[jfds71589-bib-0069] Uhrig, M. K. , N. Trautmann , U. Baumgärtner , et al. 2016. “Emotion Elicitation: A Comparison of Pictures and Films.” Frontiers in Psychology 7: 180. 10.3389/fpsyg.2016.00180. PMC4756121 26925007

[jfds71589-bib-0070] Vatral, C. D. , A. D. Gilman , and J. J. Quinlan . 2022. “Consumer Awareness of the Message Not to Wash Raw Poultry, Current Practices, and Barriers to Following That Message.” Journal of Food Protection 85, no. 6: 930–937. 10.4315/JFP-21-324. 34935942

[jfds71589-bib-0071] Wagenberg, V. , F. Brouwer , R. Hoste , and M.‐L. Rau . 2012. Comparative Analysis of EU Standards in Food Safety, Environment, Animal Welfare and Other Non‐Trade Concerns With Some Selected Countries. European Parliament.

[jfds71589-bib-0072] Waller, R. L. , and C. L. Iluzada . 2020. “Blackfish and SeaWorld: A Case Study in the Framing of a Crisis.” International Journal of Business Communication 57, no. 2: 227–243. 10.1177/2329488419884139.

[jfds71589-bib-0073] Wilcoxon, F. 1945. “Individual Comparisons by Ranking Methods.” Biometrics Bulletin 1, no. 6: 80–83. 10.2307/3001968.

[jfds71589-bib-0074] Yeung, H. F. , C. Bruhn , M. Blackburn , et al. 2019. “Evaluation of In‐Person and On‐Line Food Safety Education Programs for Community Volunteers.” Food Control 99: 34–39. 10.1016/j.foodcont.2018.12.021.

