# Supplementary material for: When Food Safety Goes Viral: The Influence of the Documentary Poisoned on Trust in Food Supply Chain Actors and Food Risk Perceptions Among Young Adult College Students in Indiana
Source: J Food Sci. 2026 Oct 21;91(10):e71589. doi: 10.1111/1750-3841.71589 (PMC13686119; doi:10.1111/1750-3841.71589)
Supplement: Supplementary file 1 [file JFDS-91-0-s001.docx]

**Supplemental Information S1**

**Screening Questionnaire**

*Information: Questions 4 and 6 were only included in Study 2.*

1. Have you watched the documentary "*Poisoned: The Dirty Truth About Your Food*" on Netflix?

- Yes
- No

2. With which gender do you identify the most?

- Male
- Female
- Prefer to self-describe: ______________
- Prefer not to answer

3. What is your age range?

- 18-24 years
- 25-34 years
- 35-44 years
- 45-54 years
- 55-64 years
- 65 years and above
- Prefer not to answer

4. What is your ethnicity? (Select what you dominantly describe as)

- American Indian or Alaska Native
- Asian
- Black or African American
- Hispanic
- Native Hawaiian or Other Pacific Islander
- White (non-Hispanic)
- Other, specify: ___________________
- Prefer not to answer

5. What do you major in? (Please, specify your field of study): ___________________
6. What is your current grade level:

- Freshman
- Sophomore
- Junior
- Senior

7. How often have you cooked in the last 6 months?

- I've cooked almost every day.
- I cook about 3-4 times a week.
- I cook once a week.
- I've cooked a few times a month.
- I've only cooked a couple of times in the last 6 months.
- I haven't cooked at all in the last 6 months.

8. First Name: __________________
9. Last Name: __________________
10. Email address: ___________________________________
