# Supplementary material for: When Food Safety Goes Viral: The Influence of the Documentary Poisoned on Trust in Food Supply Chain Actors and Food Risk Perceptions Among Young Adult College Students in Indiana
Source: J Food Sci. 2026 Oct 21;91(10):e71589. doi: 10.1111/1750-3841.71589 (PMC13686119; doi:10.1111/1750-3841.71589)
Supplement: Supplementary file 2 [file JFDS-91-0-s002.docx]

**Supplemental Information S2**

**Survey Instrument**

Participants were asked to first complete pre survey (*1. Green*) before the intervention. Immediately following the intervention, participants completed a post-survey (*2. Blue*). Responses from the pre- and post- intervention surveys were then used during the focus group. Nine days after the in-person session, participants received a follow-up survey through Qualtrics XM. The follow-up survey included the same questions administered in the pre- and post- surveys, allowing for comparison of participants’ responses across the three time points.

1. Pre-survey

| **Please give each statement a rank on a scale of how much you agree with it Scale: 1 = strongly disagree to 7 = strongly agree** | Score |
| --- | --- |
| I am confident in the U.S government’s ability to ensure safe food that won’t make me sick. |  |
| I am confident in small farmers’ ability to produce safe foods that won’t make me sick. |  |
| I am confident that large farmers produce safe food that won’t make me sick. |  |
| I am confident that small processors produce safe foods that won’t make me sick. |  |
| I am confident that large processors produce safe foods that won’t make me sick. |  |
| I am confident that restaurants produce safe foods that won’t make me sick. |  |
| I am confident that the food I purchase from the grocery store is safe and won’t make me sick. |  |
| I am confident that the food that I cook myself at home is safe and won’t make me sick. |  |

How important to you is food safety? (Circle the answer that best applies)

| 1. Not at all important | 2. Slightly Important | 3. Important | 4. Fairly Important | 5. Very Important |
| --- | --- | --- | --- | --- |

2. Post-survey

| **Please give each statement a rank on a scale of how much you agree with it Scale: 1 = strongly disagree to 7 = strongly agree** | Score |
| --- | --- |
| I am confident in the U.S government’s ability to ensure safe food that won’t make me sick. |  |
| I am confident in small farmers’ ability to produce safe foods that won’t make me sick. |  |
| I am confident that large farmers produce safe food that won’t make me sick. |  |
| I am confident that small processors produce safe foods that won’t make me sick. |  |
| I am confident that large processors produce safe foods that won’t make me sick. |  |
| I am confident that restaurants produce safe foods that won’t make me sick. |  |
| I am confident that the food I purchase from the grocery store is safe and won’t make me sick. |  |
| I am confident that the food that I cook myself at home is safe and won’t make me sick. |  |

How important to you is food safety? (Circle the answer that best applies)

| 1. Not at all important | 2. Slightly Important | 3. Important | 4. Fairly Important | 5. Very Important |
| --- | --- | --- | --- | --- |
