# Supplementary material for: When Food Safety Goes Viral: The Influence of the Documentary Poisoned on Trust in Food Supply Chain Actors and Food Risk Perceptions Among Young Adult College Students in Indiana
Source: J Food Sci. 2026 Oct 21;91(10):e71589. doi: 10.1111/1750-3841.71589 (PMC13686119; doi:10.1111/1750-3841.71589)
Supplement: Supplementary file 3 [file JFDS-91-0-s003.docx]

**Supplemental Information S3**

**Focus Group Moderator Guide**

**Introduction** **[5 minutes]**

Thanks for joining us today. I am XXXXX. I am a graduate student from the Food Safety Human Factor Lab – Feng Lab. I am here with my co-moderator(s), XXXXX who will be assisting me and taking notes during this session.

**Objectives**

During this session, we will discuss the documentary “Poisoned: The Dirty Truth About Your Food” that you just watched. Our goal is to gather insight into how this documentary impacts viewers. The information that we received from you today will help us evaluate the potential use of documentaries like this one as an educational tool. Your input is valuable in helping us understand the effectiveness and educational value of documentary style media such as Poisoned.

**Ground rules**

Before we start, I want to review a few important things.  The session will be audio-recorded because we value your opinions and thoughts, and we do not want to miss any information. There are no right or wrong answers, so please share freely. We are interested in what you have to say. I am here to ask questions, listen, and give everyone a chance to share. I will kindly request that when someone is speaking, we give them room to do so without interfering. I will also ask that if you have your phone here, put it in silent mode.  I will refer to you by name for ease, but your name will not be associated with any comments. All information will be kept confidential and encrypted. However, breach of confidentiality is always a risk with data, but we will take precautions to minimize this risk.

**Ice breaker** **[5 minutes]**

Let’s go around and introduce ourselves with your names, your major of study is at Purdue and where you are from (country or state in the U.S.).

**Personal attitude and perception of food safety. [5 minutes]**

Q1. What are your thoughts on food safety? Do you have any concerns regarding the safety of the food you consume?

*Probing: Could you elaborate on those concerns?*

*Follow-up:* *Are you doing anything at home to address your concerns?*

Q2. How would you define “Food Safety”?

Please describe in your own words what this term means to you.

**Thoughts on documentary [15 minutes]**

Q1. What are your thoughts after watching the documentary?

*Probing: Can you tell us more about how you felt when watching the documentary today?*

Q2. Did you learn something new by watching the documentary?

*Probing: Can you tell us more about what you learn*ed?

*Probing: Has anyone else learned something different from what has previously been*	*mentioned?*

Q3. Why do you think they created this documentary?

*Probing: Why do you think that?*

**Confidence in key stakeholders in the food supply chain [10 minutes]**

*“Now let’s compare your first (1. Green) and second (2. Blue) questionnaire.*

*Each participant will have their pre-survey (1. green) and post-survey (2. blue) on a sheet of paper in front of them.”*

Q1. Between your first and second questionnaires, did your answers change?

How did your score evolve for small farmers? Raise your hand if it increased (wait for participants' answer), decreased (wait for participants' answer), or stayed the same (wait for participants' answer).

Probing: Why did you make that change?

Ask the same question for small farmers, large farmers, small processors, large processors, restaurants, grocery stores, and the U.S. government.

If a participant asks to define a term, ask to “Explain what he understood when answering the pre-and post-survey”. If other participants did not have the same definition, ask them, "Now, with this information, would you have changed your answers?"

Q2. To whom did you give the **lower score** to? Why did you make this choice?

*Probing: What do you think they would need to do to score higher?*

*Who has a similar answer? Does someone else want to share their lowest score?*

Q3. Who did you give the **highest score** to? Why did you make this choice?

*Probing: Who has a similar answer? Does someone else want to share their highest score?*

Q4. Which of the outbreaks discussed in the documentary stood out to you? And why?

Probing: In your opinion, who is responsible for this outbreak?

*Follow-up (if no one responds):* Can anyone recall any outbreak presented in the documentary?

**Impact of the documentary [5 minutes]**

Q1. After watching the documentary, do you think you will change your purchasing behavior, food handling practices (at home), dining out consumption?

Q2. What information or part of the documentary will you share with your friends and family? And why?

Q3. Do you think this documentary could be used to educate about food safety?

*Probing: As a student, how do you feel about using documentaries in course?*

**End of the session** **[5 minutes]**

We have reached the end of our discussion. Thank you all for participating, I appreciate your opinions. Next week, you will receive a follow-up survey to complete online.

Before we go, is there anything you’d like to add concerning what we have discussed today?

We appreciate you taking the time out of your busy schedule to join us today.
